# Sacral Neuromodulation Outcomes for Managing Urinary Incontinence in Patients With Overactive and Neurogenic Bladder

**DOI:** 10.7759/cureus.109043

**Published:** 2026-05-17

**Authors:** Chelsae Nugent, Gabriel Carreño Galeano, Kellen Choi

**Affiliations:** 1 Department of Urology, University of Louisville School of Medicine, Louisville, USA; 2 Department of Urology, University of Louisville Hospital, Louisville, USA

**Keywords:** incomplete bladder emptying, neurogenic bladder, overactive bladder, sacral neuromodulation, urinary incontinence (ui)

## Abstract

Objective

This study aimed to assess urinary outcomes after sacral neuromodulation (SMN) implantation in patients with neurogenic bladder (NGB) and overactive bladder (OAB).

Methods

We conducted a retrospective chart review of patients who underwent permanent SNM implantation by a single surgeon between 2018 and 2025. Associated clinical encounters, operative reports, and patient-reported outcome measures were reviewed. Statistical analyses were performed using R (R Foundation for Statistical Computing, Vienna, Austria). Continuous variables were summarized using medians and interquartile ranges (IQR) and categorical variables using counts and percentages. Between-group comparisons were assessed using the Wilcoxon rank-sum test and chi-squared or Fisher's exact tests. Within-patient pre- and post-implant comparisons were analyzed using the Wilcoxon signed-rank test. Statistical significance was defined as p<0.05.

Results

One hundred and sixty-two patients who underwent permanent SNM implantation were included (NGB n=50; OAB n=112). The NGB group was younger (median age: 44.5 years; p<0.001) and had a slightly lower body mass index (BMI) (p=0.046). The OAB group was older (median age: 61.5 years) with BMI >30 and a varied sex distribution (p<0.001). Presenting symptoms differed between groups (p<0.001), with incomplete bladder emptying predominating among NGB patients and storage symptoms consistent with OAB predominating in the OAB cohort. Pre- and post-SNM comparisons indicated a decline in frequency, daytime void (p<0.001; r=0.76) and nighttime void (p<0.001; r=0.89), incontinence episodes (p<0.001; r=0.93), and daily pad usage (p<0.001; r=0.90). Despite differences in baseline characteristics and presentation, post-implant urinary outcomes were comparable in NGB patients and OAB patients (p=0.2435).

Conclusion

SNM implantation led to significant improvement in urinary symptoms in both groups. Post-implant urinary outcomes were comparable between groups despite differences in presentation. These findings suggest SNM effectiveness is better demonstrated by post-implant outcomes rather than by diagnostic category alone and support SNM as a therapeutic option across diverse bladder dysfunction etiologies.

## Introduction

Overactive bladder (OAB) is characterized by urinary urgency, often accompanied by frequency, nocturia, and urge urinary incontinence, in the absence of infection or other identifiable pathology [1,2]. It affects approximately 20% of adults worldwide, with prevalence increasing with age and obesity and disproportionately affecting women [3]. Beyond urinary symptoms, OAB is associated with impaired sleep, depression, and reduced quality of life [4-6]. Although behavioral, pharmacologic, and advanced therapies are available, long-term adherence to conservative and medical management is poor, and only a minority of patients progress to third-line interventions [7].

Neurogenic bladder (NGB) encompasses a heterogeneous group of lower urinary tract dysfunctions resulting from neurologic disease or injury, most commonly spinal cord injury, multiple sclerosis, and other demyelinating disorders [8,9]. Patients with NGB frequently experience urinary retention, incontinence, or mixed voiding dysfunction, placing them at risk for recurrent urinary tract infections, upper tract deterioration, and reduced quality of life [10]. While clean intermittent catheterization (CIC) remains a cornerstone of management, adherence can be challenging, and treatment escalation must balance symptom control with procedural invasiveness [11-13].

Sacral neuromodulation (SNM) is an established third-line therapy for refractory OAB and non-obstructive urinary retention [14,15]. Its application in patients with NGB, however, remains less well defined, and available studies are limited by small sample sizes, heterogeneous neurologic diagnoses, and variable outcome measures [16,17]. As a result, uncertainty persists regarding the effectiveness of SNM in NGB patients and whether post-implant outcomes are comparable to those achieved in patients treated for OAB.

In this study, we evaluated urinary outcomes following permanent SNM implantation in a single-surgeon cohort of patients with OAB and NGB. The primary aim was to compare post-implant urinary outcomes between these populations. We also evaluated within-patient changes in urinary symptoms following SNM to contextualize overall treatment effects. By comparing baseline characteristics and post-implant outcomes, we sought to clarify whether selected patients with NGB can achieve symptom improvement comparable to that observed in patients with OAB following SNM implantation.

## Materials and methods

Study design and patient population

We conducted a retrospective chart review of patients who underwent permanent SNM implantation by a single surgeon at University of Louisville Health in Louisville, Kentucky, between August 2018 and January 2025. The study was approved by the University of Louisville Institutional Review Board (approval number: 22.0964) with a waiver of informed consent.

Patients were included if they underwent permanent SNM implantation for OAB or NGB. NGB was defined by documented neurologic diagnoses and clinical assessment consistent with neurogenic lower urinary tract dysfunction, including multiple sclerosis, Parkinson's disease, diabetic cystopathy, idiopathic non-obstructive urinary retention, spinal cord injury, and related conditions. All remaining patients were classified as having non-neurogenic OAB. Demographic data, clinical characteristics, presenting urinary symptoms, voiding diary data (when available), operative reports, device interrogation records, and follow-up clinical documentation were reviewed.

Patients included in this study underwent permanent SNM implantation following standard clinical evaluation and decision-making consistent with institutional practice. In general, implantation was offered to patients who demonstrated clinically meaningful symptom improvement during a test phase, typically defined as ≥50% improvement in symptoms.

However, complete test phase (peripheral nerve evaluation (PNE) or staged trial) data were not available for all patients, particularly those who were initially implanted at outside institutions or who underwent device revision or replacement at our center. These patients were included if they had a documented history of prior SNM implantation with reported clinical benefit.

In patients with NGB, selection for SNM implantation was individualized, based on symptom profile, underlying neurologic condition, and clinician judgment regarding potential benefit. This reflects real-world clinical practice, where SNM may be considered in carefully selected patients despite being off-label in certain neurogenic populations.

SNM system characteristics

Implanted SNM systems included InterStim II, InterStim Micro, and InterStim X (Medtronic, Minneapolis, Minnesota, United States). InterStim II was used exclusively prior to Fall 2021, followed by mixed use with InterStim Micro through Fall 2022, after which InterStim X was used predominantly.

Outcome measures

Urinary outcomes were assessed using available patient-reported voiding diaries and detailed clinical documentation. Outcomes of interest included daytime voiding frequency, nighttime voiding frequency, number of urinary incontinence episodes, and daily pad usage. Pre-implant and post-implant values were extracted when available.

Due to the retrospective nature of the study, outcome data were not uniformly available for all patients. Voiding diary variables were included when documented in the medical record, and analyses were performed using a complete-case approach for each outcome variable. As a result, the number of observations varied across outcomes. No imputation of missing data was performed.

Post-implant outcomes were assessed at a minimum of three months following implantation or at the most recent documented follow-up, depending on data availability, consistent with real-world clinical practice.

Analyses focused on comparing post-implant urinary outcomes between OAB and NGB cohorts, with within-patient pre- and post-implant changes presented descriptively.

Statistical analysis

Continuous variables were summarized using medians and interquartile ranges (IQR) and categorical variables using counts and percentages. Between-group comparisons were performed using the Wilcoxon rank-sum test and chi-squared or Fisher's exact tests, as appropriate. Within-patient pre- and post-implant comparisons were analyzed using the Wilcoxon signed-rank test, with effect sizes calculated using rank-biserial correlation (r). Analyses were conducted using a complete-case approach, with two-sided p-values <0.05 considered statistically significant. All analyses were performed using R (R Foundation for Statistical Computing, Vienna, Austria).

## Results

Patient characteristics

A total of 162 patients who underwent permanent SNM implantation during the study period were included, comprising 112 patients with OAB and 50 patients with NGB. Baseline characteristics are summarized in Table 1. Patients in the NGB cohort were significantly younger than those in the OAB cohort and demonstrated a more balanced sex distribution, whereas the OAB cohort was predominantly female. The median body mass index (BMI) was modestly lower among patients with NGB.

**Table 1:** Baseline characteristics of implanted patients by diagnosis (with statistics) Data are presented as median (IQR) for continuous variables and number (N) with percentage (%) for categorical variables. Between-group comparisons were performed using the Wilcoxon rank-sum test for continuous variables and chi-squared or Fisher's exact tests for categorical variables. Statistical significance was defined as p<0.05. NGB: neurogenic bladder; OAB: overactive bladder; BMI: body mass index; IQR: interquartile range; UTI: urinary tract infection; MS: multiple sclerosis

Variable	NGB (n=50)	OAB (n=112)	P-value
Age, years, median (IQR)	44.5 (35.3-63.0)	61.5 (49.0-71.3)	<0.001
Female sex, n (%)	26 (52%)	93 (83%)	<0.001
BMI, kg/m², median (IQR)	29.6 (25.8-33.7)	31.2 (27.4-37.1)	0.046
Presenting symptom			
Storage symptoms (OAB), n (%)	10 (20%)	86 (77%)	<0.001
Voiding dysfunction/incomplete emptying, n (%)	29 (58%)	16 (14%)	<0.001
Mixed urinary incontinence, n (%)	0 (0%)	7 (6%)	0.18
Recurrent UTI, n (%)	5 (10%)	1 (1%)	0.01
Underlying neurologic diagnosis			
Spinal cord injury, n (%)	21 (42%)	-	-
MS/demyelinating disease, n (%)	16 (32%)	-	-
Other neurologic conditions, n (%)	13 (26%)	-	-

Presenting urinary symptoms differed substantially between groups. Patients with NGB most commonly presented with voiding dysfunction or incomplete bladder emptying, whereas patients in the OAB cohort predominantly presented with storage symptoms consistent with OAB. Within the NGB cohort, underlying neurologic diagnoses were heterogeneous and included spinal cord injury, multiple sclerosis or other demyelinating disorders, and other neurologic conditions.

Comparative post-implant urinary outcomes by diagnosis

Across the overall cohort, permanent SNM implantation was associated with significant improvement in urinary symptoms (Table 2). Paired analyses demonstrated significant reductions in daytime void frequency (Wilcoxon signed-rank test; V=149.0; p<0.001), nighttime void frequency (V=41.5; p<0.001), urinary incontinence episodes (V=16.5; p<0.001), and daily pad usage (V=25.5; p<0.001). These improvements were accompanied by large effect sizes across all outcome measures, indicating clinically meaningful symptom improvement following SNM implantation in the study population.

**Table 2:** Urinary outcomes before and after sacral neuromodulation implantation (with effect sizes) Within-patient comparisons between pre- and post-implant outcomes were performed using the Wilcoxon signed-rank test. Effect size is reported as rank-biserial correlation (r). Statistical significance was defined as p<0.05. ^†^Effect size reported as rank-biserial correlation (r)

Outcome	Pre-implant median (IQR)	Post-implant median (IQR)	n	Statistic	P-value	Effect size^†^
Daytime voids (voids/day)	10.0 (6.8-13.2)	6.5 (4.0-8.0)	56	V=149.0	<0.001	0.76
Nighttime voids (voids/night)	3.0 (2.0-4.0)	2.0 (1.0-3.0)	51	V=41.5	<0.001	0.89
Incontinence episodes (episodes/day)	6.0 (3.0-10.0)	2.0 (0.0-4.0)	38	V=16.5	<0.001	0.93
Pads per day	4.0 (2.2-6.0)	2.0 (1.0-3.0)	38	V=25.5	<0.001	0.90

Post-implant outcomes stratified by diagnosis

Post-implant urinary outcomes stratified by diagnosis are shown in Table 3. Following permanent implantation, no statistically significant differences were observed between the NGB and OAB cohorts with respect to daytime void frequency, nighttime void frequency, urinary incontinence episodes, or daily pad usage. Despite differences in baseline characteristics and presenting urinary phenotypes, patients with NGB achieved post-implant urinary outcomes similar to those observed in patients with OAB.

**Table 3:** Post-implant urinary outcomes by diagnosis: NGB versus OAB Values represent post-implant outcomes only and are presented as median (IQR). Comparisons between groups were performed using the Wilcoxon rank-sum test. Statistical significance was defined as p<0.05. n varies by outcome due to incomplete diary documentation. NGB: neurogenic bladder; OAB: overactive bladder; IQR: interquartile range

Outcome (post-implant)	NGB n	NGB median (IQR)	OAB n	OAB median (IQR)	Statistic	P-value (Wilcoxon rank-sum)
Daytime voids (voids/day)	11	6.0 (4.5-8.0)	47	7.0 (4.5-8.0)	W=234.5	0.6384
Nighttime voids (voids/night)	10	1.5 (1.0-3.0)	44	2.0 (1.0-3.0)	W=229.5	0.8373
Incontinence episodes (episodes/day)	6	2.0 (2.0-3.5)	36	2.0 (0.0-4.0)	W=117.0	0.7553
Pads/day	7	2.0 (0.5-3.5)	35	2.0 (1.0-3.0)	W=128.5	0.8501

## Discussion

SNM is an established third-line therapy for refractory OAB and non-obstructive urinary retention; however, its role in patients with NGB remains less clearly defined and is considered off-label in many clinical settings [15,16]. In this single-surgeon retrospective cohort, we found that despite substantial differences in baseline characteristics and presenting urinary phenotypes, patients with NGB achieved post-implant urinary outcomes similar to those observed in patients treated for idiopathic OAB within this cohort.

Patients in the NGB cohort were younger, more evenly distributed by sex, and more likely to present with voiding dysfunction or incomplete bladder emptying, reflecting the heterogeneity inherent to neurogenic lower urinary tract dysfunction. Nevertheless, following permanent SNM implantation, improvements in urinary frequency, nocturia, incontinence episodes, and pad usage were similar between NGB and OAB cohorts. Importantly, these improvements were not only statistically significant but also associated with large effect sizes, underscoring the clinical relevance of symptom improvement following implantation.

Prior studies evaluating SNM in neurogenic populations have suggested potential benefit but have been limited by small sample sizes, heterogeneous neurologic diagnoses, and variable outcome definitions [16-19]. Our findings extend this literature by demonstrating that, in a real-world implantation-based cohort, selected patients with NGB can achieve durable improvement in urinary symptoms comparable to that observed in patients with OAB. These results support the concept that functional urinary outcomes following SNM may be more informative than neurologic diagnosis alone when evaluating candidacy in neurogenic populations, as previously proposed [15].

From a clinical perspective, management of NGB often requires balancing symptom control with minimization of procedural burden. While CIC remains a cornerstone of therapy for many patients, adherence challenges and quality-of-life considerations frequently prompt the exploration of alternative therapies [12,13,20]. In this context, our findings suggest that SNM may represent a viable therapeutic option for selected patients with NGB who are candidates for implantation, particularly when less invasive strategies fail to provide adequate symptom relief.

This study has several important limitations. Its retrospective design introduces inherent risks of selection and information bias, particularly given the reliance on available clinical documentation and patient-reported voiding diaries. Patient selection for permanent implantation, especially within the NGB cohort, was not standardized and may favor individuals more likely to respond to therapy, thereby limiting generalizability.

Additionally, the NGB cohort was heterogeneous, encompassing multiple underlying neurologic conditions with distinct pathophysiology and potentially variable responses to SNM, which limits the ability to draw subgroup-specific conclusions. The single-surgeon, single-center experience ensures procedural consistency but may reduce external validity and reflect institution-specific patient selection practices.

Variability in sample size across outcome measures reflects incomplete data capture inherent to retrospective analyses and may affect the precision and reliability of the observed results. Differences in follow-up duration and reliance on patient-reported outcomes further contribute to potential information bias. Moreover, the absence of adjustment for potential confounders, including baseline symptom severity, age, and comorbidities, limits the ability to attribute observed outcomes directly to SNM and precludes causal inference.

Taken together, these factors, including selection bias, incomplete data capture, cohort heterogeneity, and variability in follow-up, should be considered when interpreting the findings. Accordingly, the results are best viewed as reflective of outcomes in a real-world, selected patient population rather than broadly generalizable to all patients with NGB. Although SNM remains off-label for certain neurogenic populations, its use in carefully selected patients has been increasingly reported in the literature and reflects evolving clinical practice.

Despite these limitations, the consistency of post-implant outcomes across cohorts and the magnitude of observed symptom improvement support the internal validity and clinical relevance of our findings. Following permanent implantation, no statistically significant differences were observed between the NGB and OAB cohorts with respect to urinary outcomes. While no statistically significant differences were detected, the study was not powered to identify small differences between groups. Despite differences in baseline characteristics and presenting urinary phenotypes, patients with NGB achieved post-implant urinary outcomes similar to those observed in patients with OAB within this cohort.

These findings should be interpreted within the context of a retrospective observational design and are best understood as demonstrating associations rather than causal relationships. The heterogeneity of the NGB cohort further complicates interpretation, as different neurologic conditions may respond differently to SNM, and the lack of adjustment for baseline differences limits direct attribution of comparable outcomes to the intervention itself.

While prior studies have demonstrated the effectiveness of SNM in both OAB and NGB populations, direct comparisons of post-implant outcomes between these groups using consistent outcome measures remain limited. In contrast to studies that evaluate these populations separately, our analysis focuses on a real-world implantation cohort and suggests that, among selected patients, post-implant outcomes may be similar across diagnostic categories. These findings support a more outcome-based approach to patient selection, in which response to therapy may be less dependent on diagnostic classification and more reflective of individual functional characteristics.

## Conclusions

In this retrospective cohort, permanent SNM implantation was associated with significant and clinically meaningful improvement in urinary symptoms among patients with both OAB and NGB. Despite differences in baseline characteristics and presenting urinary phenotypes, post-implant outcomes were similar between cohorts. These findings suggest that selected patients with NGB may achieve outcomes comparable to those with OAB; however, results should be interpreted cautiously given the observational design and require confirmation in prospective studies.
